# *QuickStats*: Percentage* of Adults Aged ≥18 Years Who Walked ≥10 Minutes as a Method of Transportation,^†^ by Location of Residence^§^ — National Health Interview Survey, United States, 2005, 2010, and 2015^¶^

**DOI:** 10.15585/mmwr.mm6620a9

**Published:** 2017-05-26

**Figure:**
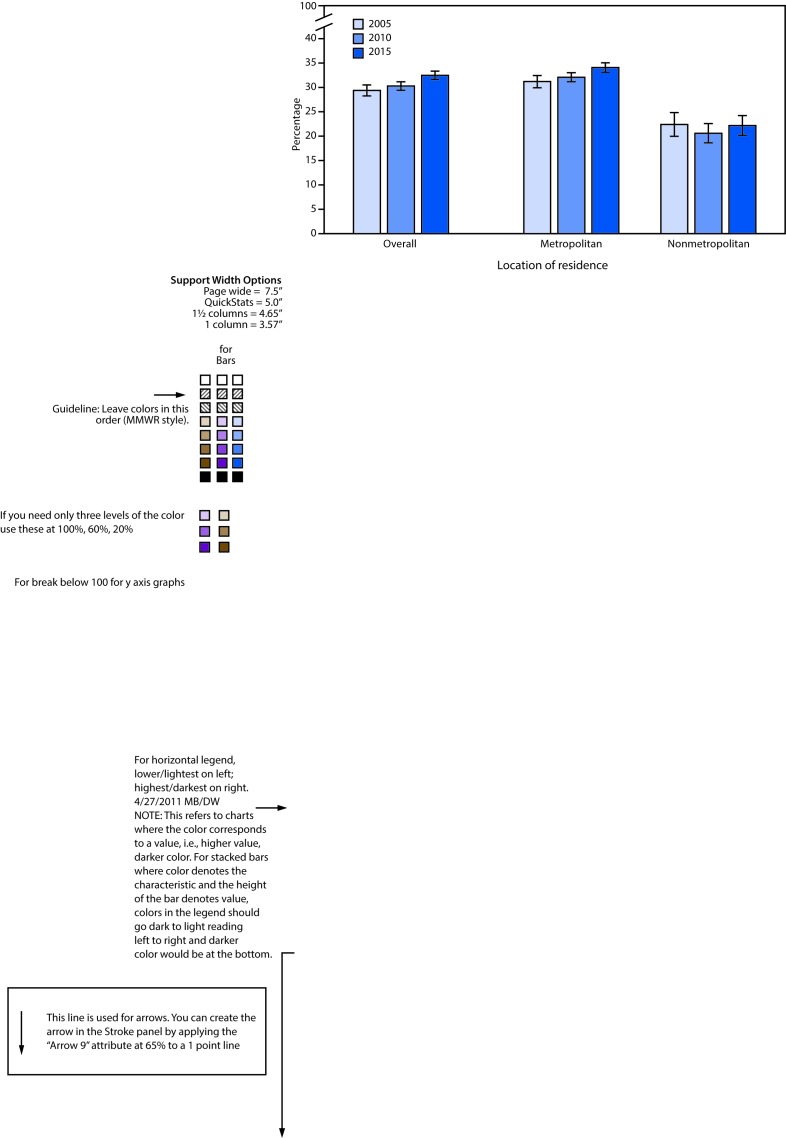
Overall, the percentage of adults aged ≥18 years that walked as a method of transportation increased from 29.4% in 2005 to 32.5% in 2015. A similar pattern was observed for adults residing in metropolitan locations (31.2% to 34.1%) but there was no change for those residing in nonmetropolitan locations (22.4% to 22.2%). Regardless of year, adults residing in metropolitan locations were more likely to have walked as a method of transportation than were adults residing in nonmetropolitan locations.

**Source:** National Health Interview Survey, 2005, 2010, 2015 data. https://www.cdc.gov/nchs/nhis.htm.

